# Breakdown of the growth–mortality trade-off along a soil phosphorus gradient in a diverse tropical forest

**DOI:** 10.1098/rspb.2023.1348

**Published:** 2023-10-11

**Authors:** Ryota Aoyagi, Richard Condit, Benjamin L. Turner

**Affiliations:** ^1^ The Hakubi Center for Advanced Research, Kyoto University, Yoshida-Konoe, Kyoto 606-8501, Japan; ^2^ Graduate School of Agriculture, Kyoto University, Kitashirakawa Oiwake-cho, Sakyo-ku, Kyoto 606-8502, Japan; ^3^ Field Museum of Natural History, 1400 S Lake Shore Dr., Chicago, IL 60605, USA; ^4^ Morton Arboretum, Lisle, IL 60532-1293, USA; ^5^ Independent researcher, Orlando, FL, USA

**Keywords:** adaptation, demography, diversity, environmental gradient, functional traits, species turnover

## Abstract

An ecological paradigm predicts that plant species adapted to low resource availability grow slower and live longer than those adapted to high resource availability when growing together. We tested this by using hierarchical Bayesian analysis to quantify variations in growth and mortality of *ca* 40 000 individual trees from greater than 400 species in response to limiting resources in the tropical forests of Panama. In contrast to theoretical expectations of the growth–mortality paradigm, we find that tropical tree species restricted to low-phosphorus soils simultaneously achieve faster growth rates and lower mortality rates than species restricted to high-phosphorus soils. This result demonstrates that adaptation to phosphorus limitation in diverse plant communities modifies the growth–mortality trade-off, with important implications for understanding long-term ecosystem dynamics.

## 1. Introduction

**Figure 1.**
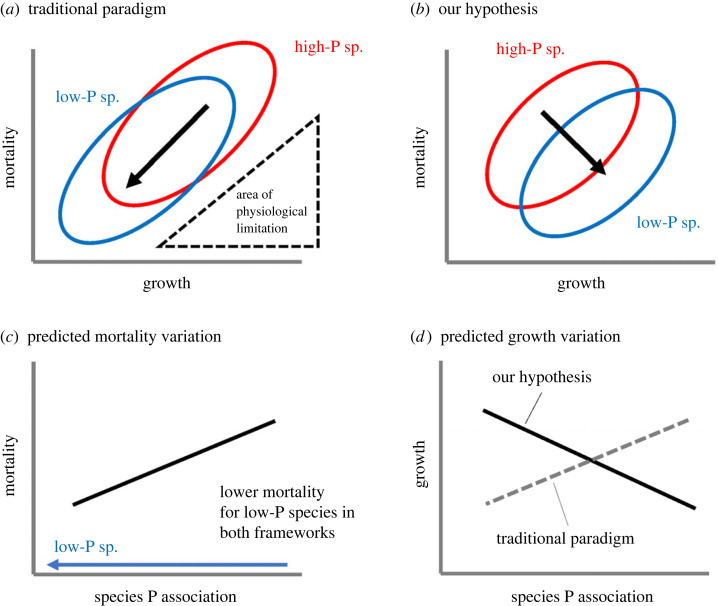
Illustrated difference between the traditional paradigm of tree species adaptation to infertile environments [3] and our hypothesis. Low-P and high-P sp. indicate plant species adapted to low-P and high-P environments, respectively. Species P association indicates the relationship of plant species distribution with a soil P gradient (i.e. species with low-P association means their distribution is restricted to low-P environments, i.e. low-P sp.). The traditional paradigm suggests that species adapted to infertile environments are selected for lower mortality rate, which is associated with lower growth rate even in infertile environments due to physiological constraints (*a, c* and *d*). By contrast, we hypothesized that tropical tree species adapted to low-P environments can enhance growth rate simultaneously while surviving longer (*b, c* and *d*).

The life-history trade-off between growth and mortality in response to variation in resource availability is a fundamental ecological concept [1,2]. In plant communities, constraints imposed by resource allocation are widely assumed to preclude species from simultaneously achieving low mortality and high growth rates, leading to the expectation that species adapted to resource-poor environments grow slower but live longer than species adapted to resource-rich environments, when growing together [3–5]. There is abundant evidence for the growth–mortality trade-off within an individual site [6,7], which mainly reflects species adaptation to different light environments. There is also evidence for the growth–mortality trade-off across soil fertility gradients at broader scale: species from infertile sites grow at a slower rate than those from fertile sites, even at low nutrient availability, and can survive longer in such environments [3,8] (figure 1*a*). However, evidence for growth–mortality trade-off in relation to soil fertility gradients comes primarily from temperate regions, where productivity is typically limited by nitrogen (N) availability [9]. By contrast, there are limited data on lowland tropical forests growing on strongly weathered soils [10], where productivity is most likely to be limited by phosphorus (P) availability [11]; many of previous studies on growth characteristic of tropical tree species adapted to infertile soils include only a small number of species [12,13], a relatively narrow range of soil P [14] or studied white-sand forests [15] where plant productivity is presumed to be limited by N rather than P [16]. Low pH and moisture availability are also suggested as limiting factors in white-sand forests [17]. Consequently, characteristics of plants growing on soils that are limited by P rather than N have not yet been fully examined.

Increasing evidence indicates that tropical tree species adapted to low-P environments (hereafter low-P species) exhibit faster growth rate than species adapted to high-P environments (hereafter high-P species) when low-P and high-P species are growing together [18,19] and that there are a number of physiological mechanisms that these low-P species use to maintain growth rate under low-P conditions [10]. For example, a meta-analysis showed that photosynthetic P-use efficiency (i.e. maximum photosynthetic capacity per foliar P concentration, PPUE) increased as foliar P concentration decreased, whereas the opposite occurred for photosynthetic N-use efficiency (N-use efficiency decreased as foliar N decreased) [20]. This difference can be explained by contrasting physiological responses of plants to P and N depletion, such that the structural fraction of foliar P (i.e. P allocated to phospholipids) decreases as foliar P decreases, resulting in high within-leaf allocation of P to photosynthetic machinery [21,22]. By contrast, the structural fraction of foliar N (i.e. N allocated to cell wall proteins) increases as foliar N declines [23,24]. Higher PPUE does not necessarily imply higher growth rate because leaves with high PPUE tend to have low P concentrations and lower maximum photosynthetic rates on a mass basis [20]. However, PPUE can be associated with fast growth on low P soils, all else being equal. For instance, leaves with a certain P concentration and high PPUE can fix more carbon than leaves with the same P concentration but low PPUE. In addition, the P concentration of non-photosynthetic organs (i.e. wood) decreases in low-P environments, leading to higher P allocation to photosynthetic organs (i.e. leaves), whereas the N concentration of wood does not decrease in N-depleted environments [25–27]. The N concentration in wood is stable across soil N gradients because N is allocated to cell walls, which cannot be reduced even when N availability is low [25]. Overall, studies on demography and physiology suggest that the current paradigm for plant adaptation to infertile environments (i.e. species adapted to infertile environments inherently exhibit lower productivity than species adapted to fertile environments) might not apply to the adaptation of trees to low-P environments.

It remains unclear how mortality rates of tropical tree species vary with fertility, because variation in species-specific mortality in relation to soil P gradients has been studied mainly in seedling experiments with a small number of species [13,25,28], while there are many studies examining the relationship between edaphic gradients and stand-level mortality (see below). The growth–mortality trade-off hypothesis predicts that the faster growth rates observed for species adapted to low-P soils in Panama [18,19] should coincide with a higher mortality rate, yet stand-level and seedling mortality rates generally decrease as soil fertility declines across tropical regions [15,28–31] (but see [13] for the opposite trend in seedling mortality). A lower mortality rate in infertile environments might be due to greater resource allocation to defence and the reduced effects of natural enemies in species growing in infertile environments [32]. These studies allow us to predict that low-P species exhibit not only faster growth rate but also lower mortality rate than high-P species when growing under equivalent environmental conditions (figure 1*b*).

In this study, we investigated how soil P affects tree demography using a hierarchical Bayesian analysis of growth and mortality of trees in long-term forest census plots across a steep gradient of soil P availability in Panama. We fitted two-level hierarchical Bayesian models, in which tree growth and mortality were modelled as a species-specific function of shade, tree size (diameter at breast height, dbh), soil P availability and moisture deficit at the first level, and each species-specific parameter (*β*_*k*, *k* = 0–4_) was modelled as a linear function of species associations for shade, P and moisture, and their interactions at the second level (*γ_k_*_,*m*; *m* = 0–7_). This approach allowed for the estimation of the species-specific effects of shade, tree size, P and water availability on tree demography, and the quantification of the extent to which those effects relate to species associations for shade, P and moisture. Specifically, we tested the hypothesis that tree species adapted to low-P environments exhibit faster growth and lower mortality rates than species adapted to high-P environments, when compared under equivalent environmental conditions. Our results challenge the paradigm that species adapted to infertile environments always exhibit low productivity in compensation for lower mortality rate.

## 2. Material and methods

### (a) Study sites and pre-data processing

This study used data collected in 43 plots across the Isthmus of Panama [33,34]. For growth analysis, we used census data for 28 817 trees, including 407 species. For mortality analysis, we used census data for 39 961 trees, including 413 species (24.5% of total trees at the initial censuses died). All trees larger than 100 mm in dbh were mapped, tagged, measured and identified to species level using previously described methods [35]. Thirty of the 43 plots included trees between 10 and 100 mm dbh, in a central 40 × 40 m quadrat. Soils varied widely across plots and included Oxisols, Ultisols, Alfisols and Inceptisols [18]. Readily available P was determined by extraction with anion-exchange membranes (i.e. resin phosphate). Several soil properties were measured for each plot, but resin phosphate was used as an index of P availability because it was strongly correlated with the distribution of 58% of 272 dominant tree species in the plot network [36]. The annual precipitation varies from 1756 to 3280 mm yr^–1^. The dry season moisture deficit, which represents the intensity of the dry season (between December and April) as the minimum annual value of cumulative daily precipitation minus evapotranspiration, varied from –392 to –580 mm. A more negative moisture deficit indicates a longer, drier, dry season. We included dry-season moisture deficit in the analyses because it was strongly correlated with the distribution of 67% of species [36] and therefore probably affected tree demography. The data for moisture and P have been previously published [18,36,37]. Palms were excluded because they rarely exhibit secondary growth.

We used the relative growth rate (RGR, mm mm^–1^ yr^–1^) of the largest or main stem as a measure of growth rate, which was calculated as follows:

$$\mathrm{RGR}=\;\frac{\ln(dbh_{t=2})\;-\ln(dbh_{t=1})}{\mathrm{census}\;\mathrm{interval}}\;,$$

where dbh_*t* = 1_ and dbh_*t* = 2_ indicate the dbh in the first and second censuses, respectively. A shading index was used as a surrogate for light availability and equalled the sum of the distance-weighted basal areas of trees larger than and within 10 m of each focal tree.

shading index= ∑BA ×exp(–α×distance),

where BA is basal area of a larger neighbour (m^2^) and α is a weighting coefficient [38]. Because shading was expected to vary with both distance and the size of neighbour trees, preliminary analyses were performed to quantify the distance decay (α) for three size classes of neighbour trees (class 1, dbh ≤ 100 mm; class 2, 100 mm < dbh ≤ 300 mm; class 3, 300 mm < dbh) separately. Larger α values indicated sharper declines in shading impact on RGR with distance. We determined the optimal value for the distance decay by comparing growth models with different combinations of α with Akaike information criterion (AIC), yielding α = 0.5, 0.2 and 0.7, for size classes 1, 2 and 3, respectively (electronic supplementary material, text S1). This indicates that medium-sized neighbours (class 2) had the strongest effects on RGR through shading. Trees within 10 m from a plot edge were excluded from all analyses because their shading indices could not be calculated.

### (b) Bayesian models for growth and mortality

We fitted a two-level hierarchical Bayesian model in which the observed RGR of individual *i* of species *j* was modelled as a species-specific function of shading, initial dbh, soil P availability and moisture deficit at the first level.

2.1 $$\begin{array}{rl} & {\mathrm{RGR}}_{\mathrm{ob}s_{i,j}}∼t({\mathrm{RGR}}_{{\mathrm{pred}}_{i,j}},\sigma ,v),\\ {\mathrm{RGR}}_{{\mathrm{pred}}_{i,j}}\;= & \;\beta_{G0_{j}}+\beta_{G1_{j}}\times \log(\mathrm{shading}+\underline{\mathrm{shading}})\\ & +\beta_{G2_{j}}\times \log(\mathrm{dbh}\;)+\beta_{G3_{j}}\times \log(\mathrm{resin}\;P)\\ & +\;\beta_{G4_{j}}\times \;\mathrm{moisture}\;\mathrm{deficit},\\ & \sigma ∼\mathrm{uniform}\left(\frac{1}{1000}\right),\\ & v∼\exp\;\left(\frac{1}{29}\right)+1\;, \end{array}$$

where the parameters *β*_*G* 0–4*j*_ describe the ‘intrinsic' growth rate [39] and growth response to shading, size, soil P and moisture deficit of species *j*, respectively. The predicted RGR at a given shading, size, P availability and moisture deficit was modelled with a *t*-distribution including ‘scale' (*σ*) and ‘normality' (*ν*) parameters because it allows robust statistical estimation for data with outliers [40]. The scale parameter *σ* is comparable to the standard deviation of the normal distribution. Normality parameter *ν* describes the tails of the *t*-distribution. If *ν* = 1, the *t*-distribution has fat tails, whereas if *ν* greater than 30, the distribution is almost identical to a normal distribution. The prior of *v* was assumed to be exponentially distributed with a mean of 30, which gave equal probabilities for values greater or less than 30 [40]. In our analysis, *ν* was estimated at 2.16 (95% confidence interval (CI), 2.09–2.23), which indicates that the data included outliers. Before log-transformation, a constant value (the median value of the shading index denoted as shading in equation (2.1)) was added to the shading index of each tree to avoid zero values (i.e. a tree with no larger neighbouring trees within 10 m). For simplicity, we excluded interactions among the environmental factors (see electronic supplementary material, text S2 for further discussion).

Mortality analysis is complicated by differences in census intervals among the plots. The census intervals varied from 3 to 16 years (greater than 10 years for 31 plots). To overcome this limitation, we developed a two-level hierarchical Bayesian model that incorporated census intervals. Tree status (dead or alive) was modelled using a Bernoulli probability function with the probability of mortality, *p*_mortality *i*,*j*_, for individual *i* of species *j*. We assumed that 1 – *p*_mortality *i,j*_ (i.e. the probability of survival) decreases with census interval (units equal to years) following a power function of 1 – annual mortality rate (i.e. annual survival rate). The annual probability of mortality was fitted using a logistic link function. The model equations for mortality are as follows:

2.2 $$\begin{array}{rl} {\mathrm{status}}_{i,j}\; & (1,\mathrm{dead};0,\mathrm{alive})∼\mathrm{Bernoulli}\;(\;p_{{\mathrm{mortality}}_{i,j}}),\\ p_{{\mathrm{mortality}}_{i,j}}\;= & \;1-\;{\left(1-\;p_{\mathrm{annual}\;{\mathrm{mortality}}_{i,j}}\right)}^{\mathrm{census}\;\mathrm{interval}},\\ p_{{\mathrm{annual}\;\mathrm{mortality}}_{i,j}}=\; & \mathrm{logistic}[\beta_{M0_{j}}+\beta_{M1_{j}}\times \log(\mathrm{shading}+\underline{\mathrm{shading}})\\ & +\beta_{M2_{j}}\times \log(dbh\;)+\beta_{M3_{j}}\times \log(\mathrm{resin}\;P)\\ & +\;\beta_{M4_{j}}\times \;\mathrm{moisture}\;\mathrm{deficit}], \end{array}$$

where parameters *β_M_*_0–4*j*_ describe the ‘intrinsic’ mortality rate and mortality response to shading, size, soil P and moisture deficit of species *j*, respectively, as in the growth model.

At the second level of the hierarchical growth and mortality models, the species-specific parameters from the first level, *β_G kj_* (*k* = 0–4) or *β_M kj_* (*k* = 0–4), were modelled as a linear function of species environmental associations, and non-informative priors were set for *σ_k_* and *γ_k_*_,*m*_.

$$\begin{array}{rl} & \beta_{Gk_{j}}or\;\beta_{Mk_{j}}∼\mathrm{normal}\;{\left(\mu_{k},\sigma_{k}\right)}_{k=0,1,2,3,4}\;,\\ \mu_{k}= & \;\gamma_{k,0}+\gamma_{k,1}\times P\;\mathrm{association}+\;\gamma_{k,2}\;\times \mathrm{shade}\;\mathrm{association}+\;\gamma_{k,3}\\ & \times \mathrm{moisture}\;\mathrm{association}\;+\;\gamma_{k,4}\times P\;\mathrm{association}\;\\ & \;\times \mathrm{shade}\;\mathrm{association}+\;\gamma_{k,5}\times P\;\mathrm{association}\\ & \;\times \mathrm{moisture}\;\mathrm{association}+\;\gamma_{k,6}\;\\ & \times \mathrm{shade}\;\mathrm{association}\times \mathrm{moisture}\;\mathrm{association}+\;\gamma_{k,7}\\ & \times \mathrm{interaction}\;\mathrm{between}\;\mathrm{three}\;\mathrm{associations},\\ & \gamma_{k,m\;k=0,1,2,3,4;m=0,1,2,3,4,5,6,7\;}∼\;\mathrm{normal}(0,0.01).\\ & \sigma_{k\;k=0,1,2,3,4}\;∼\;\mathrm{uniform}\left(\frac{1}{1000}\right). \end{array}$$

Species P and moisture associations were determined in a previous study [36] as the slope of the response of species occurrence frequency to soil P and moisture deficit, respectively. Species with lower P association (effect size less than 0; i.e. species adapted to low P) had a negative relationship between occurrence frequency and soil P availability, whereas species with higher P association (effect size greater than 0; i.e. species adapted to high P) had a positive relationship between occurrence frequency and soil P availability.

We determined shade association as follows:

$$\begin{array}{ll} & \;\;\text{shade association of a species}\\ & =\mathrm{median}\;\left(\begin{array}{c} \text{ shade index of each individual}\\ \text{–}\text{median shade index for each}\\ \text{individual's site} \end{array}\right) \end{array}$$

Shade association scores were standardized using site-specific median shade indices because tree density, which strongly influences the shade index, varied across plots. Electronic supplementary material, figures S2, S3 and S4 present correlations between traits (maximum dbh, wood density, and foliar N and P concentrations) and association with shade and soil P, and among-plot variation in tree density and shading index, respectively. The trait data were obtained from a 50 ha plot on Barro Colorado Island [7] where the soil has an intermediate P availability.

The posterior distributions of the model parameters were estimated using the Gibbs sampling method of the Markov chain Monte Carlo approach (MCMC). MCMC calculations were performed using R and Jags v. 4.3.0 [41]. We monitored convergence by running three chains with different initial values and used Gelman and Rubin's convergence diagnostics (performed with the coda package in R) and a value of 1.1 to detect convergence [42]. Convergence required less than or equal to 250 000 iterations; therefore, we used a burn-in period of 300 000 iterations and an additional 10 000 iterations for analysis. The R code is available in the Dryad Data Repository [43].

We examined the relationship between species environmental association and species-specific parameter *β_kj_* of the growth and mortality models (i.e. *γ_k_*_,*m*_; *k* = 0–4, *m* = 0–7) to capture the effects of species-level environmental associations on interspecific variation in responses to size and environmental factors (*β*_1–4*j*_). The average effects of size and environmental factors were assessed both by the intercept of the relationship (*γ_k_*_,0_; *k* = 1–4) and visual inspection of the trend in most species. The degrees of significance for intercepts and slopes were tested using 95% CIs.

We further calculated species growth and mortality rates under infertile, intermediate and fertile environmental conditions (5th, 50th and 95th quantiles of resin P, respectively = 0.2, 2.3 and 12.9 mg P kg^–1^) and intermediate moisture and shade levels (50th quantile values) at 100 mm dbh using Bayesian models. We determined how the simulated growth and mortality for each species were related to species association with shade, soil P and moisture with an abundance-weighted linear regression analysis using the lm function in R. Species with greater than or equal to 10 individuals and greater than or equal to 100 mm dbh maximum size were used for the analysis. Mortality rate was log-transformed to meet the assumption of normality. Model selection was performed using the step function in R to exclude variables with small effects. The relationship between growth and mortality and how the relationship differs depending on species association with soil P were also examined using a regression approach to understand the trade-off between high growth and low mortality when species group associated with soil P availability was considered separately.

## 3. Results

### (a) Effects of environmental factors on tree growth and mortality and their relationship with species P association

**Figure 2.**
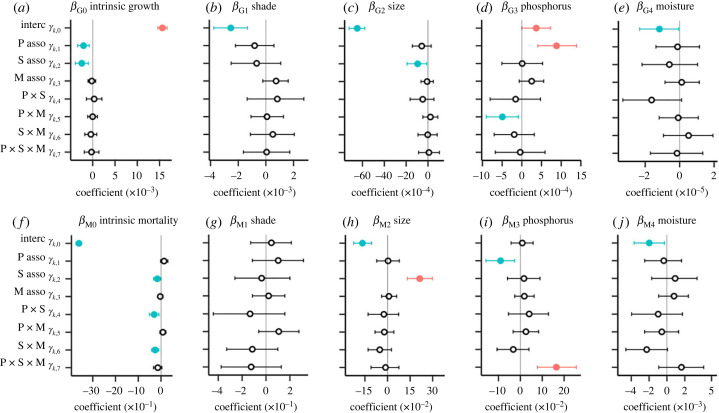
Effects of environmental variables and size on tree growth and mortality (*β_Gk_* and *β_Mk_*) and their relationships with species environmental associations (*γ_Gk_* and *γ_Mk_*), estimated by the first and second level of hierarchical Bayesian modelling, respectively. *β_G_*_0_ and *β_M_*_0_ (*a*,*f*) indicate species-level intrinsic demographic rates. *γ* values involve an intercept (denoted as interc; *γ*_k,0_), single effects of species associations for P, shade and moisture (denoted as P-asso, S-asso and M-asso, respectively; *γ*_k,1–3_), and interactions among the species environmental associations (*γ*_k,4–7_). Circles and error bars indicate the median value and 95% confidence intervals calculated by the posterior simulations, respectively. An open circle indicates that the confidence interval includes zero, while a filled circle indicates that the confidence interval ranges under (blue) or over (red) zero. A significant intercept of an environmental factor (*γ_k_*_,0; *k* = 1–4_) indicates a significant impact of the factor on either growth or mortality for most species. For example, red filled circles of *γ*_3,0_ for *β_G_*_3_ (*d*) indicate that the variation of soil P positively affects growth rate on average (electronic supplementary material, figure S5A).

**Figure 3.**
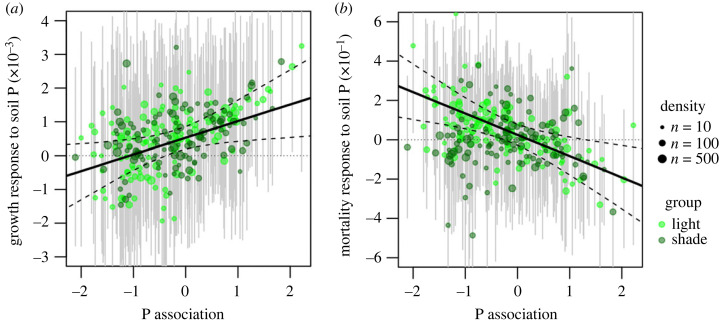
The response of growth (*a*) *β*_G3_ (see equation (2.1) in Material and methods) and mortality (*b*) *β*_M3_ (see equation (2.2) in Material and methods) to natural variation in soil P among tree species differing in P association (i.e. whether a species tends to occur at low or high P availability). The responses to growth and mortality are predictions from species-specific Bayesian models, with positive values indicating an increase in the variable with increasing soil P availability. Each dot represents the predicted growth or mortality response of an individual species, and the size of dots shows tree density. Species with shade association lower (i.e. light-demanding) and higher (i.e. shade-tolerant) than the median shade association (0.14) are indicated with light and dark green symbols, respectively. Error bars represent 95% confidence intervals obtained by posterior simulations. The slope (*γ*_3,1_ + *γ*_3,4_ + *γ*_3,5_ + *γ*_3,7_) and intercept (*γ*_3,0_ + *γ*_3,2_ + *γ*_3,3_ + *γ*_3,6_) of the black line were calculated for the median value of shade association and zero moisture association (i.e. moisture generalist). Dashed black lines show 95% confidence intervals, calculated as the 2.5th and 97.5th quantiles of predictions from posterior simulations for species with median shade association and zero moisture associations.

All explanatory variables in the models, including shade, tree size, soil P availability and moisture deficit, significantly affected either the growth or mortality rates of tree species (*γ_k_*_,0_, figure 2). The growth of an average tree predicted by the hierarchical model increased slowly but significantly with increasing soil P availability (*γ_G_*_3,0,_ figure 2*d*; electronic supplementary material, figure S5A). Indeed, the growth of most species (73%) responded positively to soil P (figure 3*a*). By contrast, mortality rate did not vary significantly with soil P availability (electronic supplementary material, figure S5B). The intercept of the relationship between species environmental association and mortality response to soil P availability was not significantly different from zero (*γ_M_*_3,0_, figure 2*i*), indicating that, on average, the effect of soil P availability on mortality was small in this tree community.

The magnitude of species-specific growth (figure 3*a*; figure 2*d*, *γ*_G3,1_) and mortality (figure 3*b*; figure 2*i*, *γ_M_*_3,1_) responses to an increase in soil P availability were significantly correlated with species P association. An increase in P availability caused a greater growth response for high-P species than for low-P species. By contrast, an increase in P availability caused a greater increase in mortality for low-P species than for high-P species, indicating the sensitivity of low-P species to increasing soil P.

Overall, 83% of the species had a negative growth response to shade (electronic supplementary material, figure S1B), and the intercept of the relationship between species environmental association and growth responses to shading was significantly different from zero (*γ_G_*_1,0_, figure 2*b*). This indicated that the tree growth rate decreased as shading increased; however, shading had little impact on the mortality rate: the intercept of the relationship between species environmental association and mortality responses to shading was not significantly different from zero (electronic supplementary material, figure S1F; *γ_M_*_1,0_, figure 2*g*). Growth and mortality rates decreased when the site was wetter (i.e. sites with less negative moisture deficit), because the demographic response of most species to moisture deficit was negative for growth and mortality (71% and 61% of all species, respectively; electronic supplementary material, figures S1D and H, and figure 2*e* and *j*).

### (b) Interspecific variations in growth and mortality at a standardized environmental condition and growth–mortality trade-off

**Figure 4.**
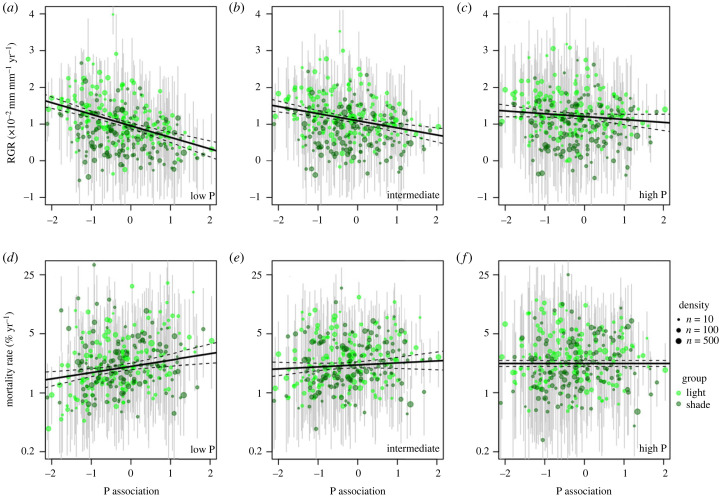
Relationships between species association with soil P and simulated relative growth rate (RGR) (*a,b*,*c*) and annual mortality rate (*d,e*,*f*), weighted by species abundance, at 100 mm diameter at breast height (dbh). Only species with greater than or equal to 10 individuals and greater than or equal to 100 mm dbh maximum size were used for the analysis. RGR and annual mortality rate were estimated at 5th, 50th and 95th quantiles (low-P, intermediate and high-P conditions, respectively) of resin P with median shading and moisture levels by the Bayesian models using the data from 43 plots in Panamanian forests (see electronic supplementary material, figure S6 for the habitat ranges of low-P and high-P species). Each dot indicates the RGR or mortality of a species and the dot size shows tree density. Error bars are 95% confidence intervals. Species with shade association lower (i.e. light-demanding) and higher (i.e. shade-tolerant) than the median value (0.14) are indicated with light and dark green symbols, respectively. Slope and intercept were calculated by multiple linear regression analysis (electronic supplementary material, table S1). Dashed lines show 95% confidence intervals.

**Figure 5.**
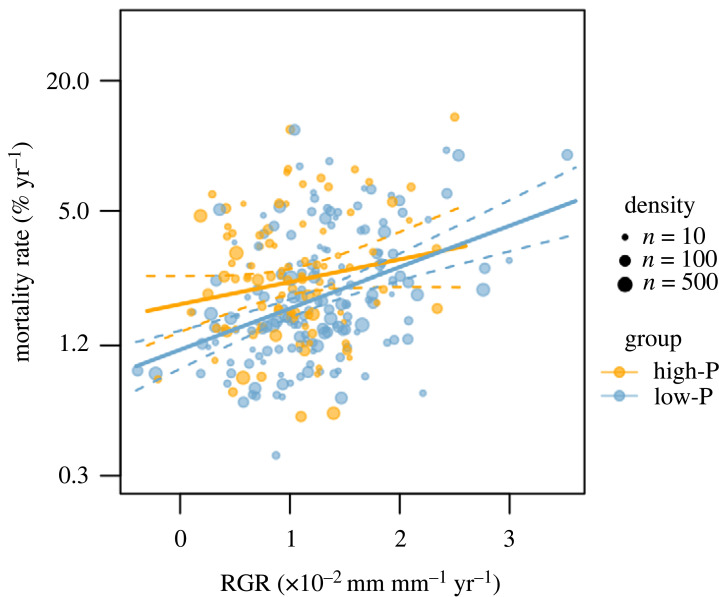
Relationship between simulated relative growth rate (RGR) and annual mortality rate, weighted by species abundance for trees of 100 mm diameter at a breast height (dbh). RGR and annual mortality rate were estimated at median shading, resin P and moisture levels by the Bayesian models fitted to the data from 43 plots in Panamanian tropical lowland forests. Species with greater than or equal to 10 individuals and maximum size greater than or equal to 100 mm dbh were used for the analysis. Each dot indicated simulated RGR and annual mortality rate of a species, and the size of dots show tree density. Species with P association lower than (low-P) and higher than zero (high-P) were indicated with blue and orange symbols, respectively. Slope and intercept of regression lines were calculated for the two groups separately with the lm function in *R* (*R*^2^ = 0.17, *p* < 0.001 and *n* = 169 for low-P group; *R*^2^ = 0.02, *p* = 0.07 and *n* = 97 for high-P group). Dashed lines show 95% confidence intervals. Note that the positive relationship for each species group indicates the growth–mortality trade-off exists within a site while our data does not support growth–mortality trade-offs across sites (i.e. among species with different P associations) (figure 4).

Consistent with our hypothesis, low-P species exhibited faster growth rates and lower mortality rates than high-P species under infertile and intermediate environmental conditions (5th and 50th quantile resin P, respectively; figure 4; electronic supplementary material, table S1), despite low-P species having lower foliar nutrient concentrations than high-P species (electronic supplementary material, figure S2C and S2D). As explained above, an increase in P availability caused a greater growth response for high-P species than for low-P species (figure 3*a*). However, low-P species exhibited higher growth rates than high-P species even under fertile environmental conditions (95th quantile resin P; figure 4*c*) although the growth advantage of low-P species was smaller at high fertility than at low fertility (figure 4*a*,*b*). On the other hand, the mortality advantage of low-P species disappeared when mortality was estimated under fertile conditions (95th quantile resin P; figure 4*f*; electronic supplementary material, table S1). There was a significant or marginally significant growth–mortality trade-off when species group associated with soil P availability was considered separately (figure 5). Species with low shade association (i.e. light-demanding species) tended to exhibit greater RGR and mortality rates than species with high shade association (figure 4; electronic supplementary material, table S1).

## 4. Discussion

We found that low-P species exhibited faster growth rates and lower mortality rates than high-P species under intermediate and infertile environmental conditions. Growth declined and mortality increased with decreasing soil P availability for high-P species, while growth declined less and mortality even decreased for low-P species. These results indicate that low-P species exhibit faster growth rate and lower mortality rate than high-P species at least in the part of the P gradient where they naturally occur (i.e. on relatively low-P soils). High-P species paradoxically do not show a growth or mortality advantage even under fertile conditions. This raises the question of how species turnover is maintained across the P gradient. Although this remains unknown, we speculate that the inability of low P species to colonize high P soils is linked to a trade-off in reproduction [10], or to the inability of low P species to downregulate investment in P acquisition mechanisms [19].

The result that species growing naturally on nutrient-depleted soils simultaneously achieve high growth and low mortality rates challenges the paradigm that species adapted to infertile environments have been selected for lower mortality at the expense of slower growth [3,4]. Studies on tree seedlings have demonstrated a similar result. A study of seedlings of light-demanding species from the Panamanian lowlands showed that tree species adapted to low-P grew faster than those adapted to high-P when grown under low-P condition [19]. Similarly, palm species from infertile sites grew faster than species from fertile sites in a transplant experiment at an infertile site in lower montane forest in western Panama [28]. By contrast, in a comparison of the performance of 660 tree species at Lambir Hills Park, Malaysia, species naturally growing in the most infertile soil exhibited the slowest growth rate even when growth was compared on the infertile soil [14]. However, there is relatively little variation in available P across the soil types at Lambir Hills [44], particularly in comparison with the 100-fold variation in resin P across our P gradient [18,36].

A possible explanation for the inconsistency between our results and the growth–mortality paradigm is that development of the growth–mortality theory was based on studies in regions where productivity is limited primarily by N rather than P. Multiple mechanisms can enhance the growth rate of trees growing on low-P soils [10], including greater allocation of P to photosynthetic machinery through a decrease in allocation to phospholipids [22,45] and non-photosynthetic organs [25,26], or increasing P-uptake capacity by (1) enzymes that allow the plant to acquire P from organic compounds [18,46,47] and (2) carboxylates that release P from sorption sites on soil surfaces [48]. On the other hand, wood density, which is often negatively correlated with mortality rate among coexisting species [7,49–52], tended to be lower for low-P species (electronic supplementary material, figure S2), suggesting that lower mortality of low-P species must be explained by something other than wood density. Some studies showed that conservative traits such as higher allocation to below-ground biomass [28,53–55] and higher allocation to defence [32,56] and carbon storage [57–59] were associated with lower mortality. These conservative traits may decrease resource losses caused by leaf turnover, the attack of natural enemies and fallen debris [60]. We do not have evidence for the relationship between traits associated with mortality and the growth-related mechanisms in low-P environments, but if those traits are not in a trade-off relationship under P-limiting conditions, then physiological mechanisms that enhance growth can explain why low-P species in Panama can exhibit faster growth and lower mortality than high-P species.

Despite low-P species having overall faster growth and lower mortality than high-P species, we detected a significant or marginally significant growth–mortality trade-off when each group was considered separately. This suggests that a growth–mortality trade-off should be observed at an individual site where species with a similar P association and contrasting shade associations coexist, but not across a fertility gradient where the community composition transitions from low- to high-P species. Within-site growth–mortality trade-offs have been demonstrated in tropical forests [6], and light-demanding tree species in our plots exhibited faster growth and mortality rates than shade-tolerant species. In agreement with this, Baltzer & Thomas [61] found a second axis in the leaf economic spectrum related to foliar P concentration across Bornean tree species growing on different geological substrates, where the first axis primarily represents the shade-tolerance continuum. These results indicate that the growth–mortality relationships differ among species occupying different eco-physiological spaces (P association versus shade association). This questions the generality of the trade-off paradigm across fertility gradients in tree communities on low P soils, indicating that the trade-off applies only to groups of species under specific environmental conditions.

### (a) Environmental effects on intraspecific variation in tree demography

Soil P availability can influence the intraspecific variation in growth and mortality rates of tree species either directly or indirectly. A direct effect of soil P was detected for growth because the growth response to increasing soil P availability was positive for most species. This is consistent with widespread P limitation in lowland tropical forests [18,62]. It has been suggested that the effect of soil P availability on tree performance involves indirect consequences of light availability. Trees compete for belowground resources rather than light on infertile soils, resulting in relatvely high light availability [63]. However, we found the opposite trend for the relationship between soil P availability and light regimes: trees were slightly more shaded on low-P soils at 10–100 mm dbh (*p* = 0.12, electronic supplementary material, figure S4), which might be related to the lower mortality and faster growth rate of low-P species. Shading decreased tree growth, confirming that competition for light is an important factor limiting forest dynamics [64–66]. Decreasing soil P availability therefore possibly negatively affects growth of tropical trees not only via direct effects on growth, but also via decreasing light availability due to more shaded conditions in forest understorey.

In contrast with growth response, the mortality response to soil P availability was small and varied in relation to species P association. A greater mortality rate in fertile environments is common for adult trees at the plot level in tropical forests [30,31]. However, positive [28], negative [13] and mixed [67,68] effects of soil fertility on intraspecific variation in mortality rate have been reported for tropical tree seedlings. Our results suggest that the inconsistency might be partly explained by the soil P associations of the species under study.

Moisture deficit is an important control on tree species distributions [36] and the growth and mortality rates of small trees [18,69] in Panama. Here, we found that growth and mortality rate both increased at drier sites (i.e. the growth and mortality responses to moisture deficit were negative for most species). Drier conditions might increase growth rate by either increasing light availability [65] or decreasing the attack of natural enemies [70]. However, this effect may change in relation to tree size: the effect of moisture deficit on growth was positive when the interaction between tree size and moisture was included (electronic supplementary material, table S5), as in the previous study in Panama [18]. This means that small and large trees respond differently to drier conditions, presumably because smaller trees suffer greater water stress than adults due to a less developed root system. Furthermore, El Niño-driven drought decreases growth and increases mortality of canopy trees in the tropics [71], suggesting that sudden and consistent precipitation fluctuation may have different effects on plant growth. Although the aim of this study was not to examine the effects of dry climate on tree performance, our results suggest that the dry–wet continuum of tropical regions influences forest structure via its effect on tree demography.

### (b) Implications for long-term ecosystem dynamics

The finding that tropical trees simultaneously achieve faster growth and lower mortality on infertile soils redefines our understanding of the controls over tree demography and ecosystem dynamics in tropical forests and potentially in plant communities on low-P soils elsewhere [3,4]. Biogeochemical theory predicts that progressive P depletion associated with long-term pedogenesis [72] drives a decline in tree productivity and forest biomass as soils age (a process termed ‘retrogression'), at least in species-poor forests [73]. Although most tropical soils are strongly weathered, there is little evidence of retrogression (i.e. the decline of productivity over long-term ecosystem development) in species-rich tropical forests [74]. We propose that high tree diversity in the tropics, supported by high species turnover along soil P gradients, is a key mechanism explaining the breakdown of the growth–mortality trade-off and the absence of retrogression [18], because diverse tropical tree communities contain species with physiological adaptive mechanisms that allow them to maintain fast growth and low mortality, even on extremely infertile soils. We expect that this phenomenon occurs widely, given the prevalence of diverse plant communities on low-P soils worldwide [75,76].

## Data Availability

Data on tree demography, species traits, site environmental characteristics and R codes used for the Bayesian analyses have been published previously (Condit *et al*., *Dryad, Dataset* (2019), http://dx.doi.org/10.15146/mdpr-pm59 [33]; Condit *et al*., *Center for Tropical Forest Science Databases* (2016), http://dx.doi.org/10.5479/data.stri.2016.0622 [34]; Wright *et al.*, *Ecology*. **91**, 3664–3674 (2010) [7]; Turner and Condit, *Dryad, Dataset* (2022), http://dx.doi.org/10.7291/D1B963 [37]; Aoyagi, *Dryad, Dataset* (2023), http://dx.doi.org/10.5061/dryad.q83bk3jpp [43]). Growth and mortality values of each species are provided as electronic supplementary material [77].
